# A portrait of single and multiple HPV type infections in Brazilian women of different age strata with squamous or glandular cervical lesions

**DOI:** 10.1186/1471-2334-14-214

**Published:** 2014-04-22

**Authors:** Leandro Santos de Araújo Resende, Sílvia Helena Rabelo-Santos, Luís Otávio Sarian, Rosane Ribeiro Figueiredo Alves, Andréa Alves Ribeiro, Luiz Carlos Zeferino, Sophie Derchain

**Affiliations:** 1 Department of Obstetrics and Gynaecology, Faculty of Medical Sciences, State University of Campinas – UNICAMP, Campinas, SP, Brazil; 2 School of Pharmacy, Federal University of Goiás, Goiânia, GO, Brazil; 3 Department of Medicine, Catholic University of Goiás, Federal University of Goiás, Goiânia, GO, Brazil

**Keywords:** Human Papillomavirus, Cervical intraepithelial neoplasia, Genotype, Prevalence, Age

## Abstract

**Background:**

Cervical cancer ranks third in prevalence and fourth as cause of death in women worldwide. In Brazil, 17,540 women were diagnosed in 2012 with the disease. Persistent infection with high-risk HPV types is a necessary condition for the development of pre-invasive and invasive cervical neoplasia. Currently, over 100 HPV types have been identified, but HPV16 and 18 are recognized as the mayor culprits in cervical carcinogenesis. Our objective was to assess the relationships between single- (ST) and multiple-type (MT) HPV infections with patients’ age and lesion pathological status.

**Methods:**

328 patients with either squamous or glandular intraepithelial or invasive cervical lesion were selected. All subjects were tested for HPV genotypes with reverse hybridization for 21 high- (hr-HPV) and 16 low-risk (lr-HPV) probes. Prevalence of ST and MT HPV infections was compared across histological types and age strata.

**Results:**

287 (87%) women had at least one HPV type detected and 149 (52%) had MT infections. The most prevalent HPV type was HPV16, present in 142 cases (49% of all HPV-positive cases), followed by HPV58, 52, 31, 35 and 33. HPV18, in single or multiple infections, occurred in 23 cases (8% of hr-HPV cases). Almost all glandular lesions were associated with HPV16 and 18 alone. Multiple infections were significantly more prevalent in squamous than in glandular lesion for HPV16 and 18 (P = 0.04 and 0.03 respectively). The prevalence of MT infections followed a bimodal distribution; peaking in women younger 29 years and in those aged 50 to 59.

**Conclusions:**

Our data indicate that prevention strategies for pre-invasive and invasive squamous lesions should be focused on HPV16 and a few alpha-9 HPV types. It is clear to us that in young women, prophylaxis must cover a large amalgam of HPV types beyond classic HPV16 and 18.

## Background

Cervical cancer is the third most prevalent cancer worldwide, being recognized as the fourth cause of death due to cancer in women. It is the second more prevalent type of cancer in women 44 years old or younger [1]. Brazilian estimates for 2012 show that roughly 17,540 women will be diagnosed with the disease in the country [2], with an estimated risk of 17 cases in 100,000 women.

High-risk human Papillomavirus (hr-HPV) persistent infection is considered the causal factor for pre invasive and invasive cervical carcinoma [3]. More than 100 HPV types have been identified and 40 are sexually transmitted [4]. High-grade intraepithelial lesion and invasive carcinoma, either squamous or glandular, are mostly associated with oncogenic HPV types included in alpha-9 (HPV 16, 31, 33, 35, 52, 58 and 67) and alpha-7 (HPV 18, 39, 45, 59, 68 and 70) species, considering that the types belonging to a species have 80% of genetic similarity [5]. Among these types, HPV 16 and 18 infections, followed by HPV 31 and 45 are found in more than 80% of cervical cancer specimens [6-9]. The prevalence of alpha 7-HPV types is lower than that of alpha-9 types, but HPV types from both species are associated with stable and persistent infections [10]. In healthy women, the prevalence of hr-HPV follows a bimodal distribution, peaking in women aged 20 to 24 years and in those aged 50 to 54; multiple-type (MT) HPV infection is more prevalent in women 30 years old or younger [11].

Single type (ST)-HPV 16 and 18 infections have been unequivocally linked to high-grade lesions (either cervical intraepithelial neoplasia (CIN) or *in situ* adenocarcinoma (AIS)) and invasive squamous/glandular cervical cancer (CC). MT-HPV has been observed in women with persistent infections, but its relationship with cervical carcinogenesis has not been established [12]. Importantly, there is no consensus whether MT-HPV infections harbouring HPV16/18 and other HPV types are associated with higher risk of carcinogenesis than ST-HPV16/18 infections [13]. In this study, we assessed the prevalence of ST and MT-HPV infections in women with squamous and glandular lesions of different age strata. Our objectives were to evaluate whether ST and MT differed 1) in its association with the type of the cervical lesion and 2) according to the patients age.

## Methods

Three-hundred twenty-eight Brazilian women with CIN, AIS, squamous or glandular CC were included. Diagnoses were obtained with diathermic conization (either LEEP or LLETZ). The women included in this study were treated in two different urban centres: 118 cases at the Department of Obstetrics and Gynaecology, Faculty of Medical Sciences, State University of Campinas – UNICAMP, Campinas, SP, Brazil and 210 at the Department of Obstetrics and Gynaecology, Federal University of Goiás, Goiânia, GO, Brazil. All women first attended a primary screening program and were referred to the institutions aforementioned because they had abnormal cytological (Pap smear) results. Pap smear results were classified as: inflammatory, squamous and glandular lesions. The visit at the referral institutions comprised colposcopy and collection of a second Pap test. This study was approved by the respective Institution’s Internal Review Boards of the two institutions (Comitê de Ética em Pesquisa da Santa Casa de Misericórdia de Goiânia, Goiânia, Goias, Brazil, and Comitê de Ética em Pesquisa da Faculdade de Ciências Médicas da Unicamp, Campinas, São Paulo, Brazil) and all selected women voluntarily signed an informed consent form prior to enrolment.

### Sample processing and DNA extraction

The cervical specimens for HPV-DNA were obtained with an endocervical brush, and stored in a 1-mL tube containing Specimen Transport Medium (STM, QIAGEN Biotechnology Brazil Ltd.). Aliquots of 200 mL of the STM were centrifuged for 10 minutes at 13,000 g. Supernatants were immediately removed and the cellular pellets were split into two parts and stored at -80°C until re-suspension in 200 μL of digestion solution (1 mM Tris, 200 mg of proteinase K/μL, and 0.5% sodium dodecyl sulphate). Digestion was performed at 55°C for 2 hours and was followed by a 5-minute incubation at 95°C to inactivate proteinase K. Nucleic acids were then purified by phenol-chloroform extraction followed by ethanol precipitation. After the DNA pellet had dried, it was dissolved in 100 μL of Tris/EDTA (1 mM and 100 μM, respectively, pH 8.2).

### HPV-DNA testing

HPV DNA of samples was amplified using the Linear Array HPV Test (LA, Roche Molecular Diagnostic, Pleasanton, USA) for genotyping, based on the principle of DNA amplification by Polymerase Chain Reaction (PCR). This test detects 21 hr-HPV probes (hr-HPV 16, 18, 26, 31, 33, 35, 39, 45, 51, 52, 53, 56, 58, 59, 66, 67, 68, 69, 73, 82 and IS39) and 16 low-risk-HPV probes (HPV6, 11, 40, 42, 48, 54, 55, 57, 61, 62, 70, 71, 81, 83, 84 and CP6108). The amplification profile was: activation of AmpliTaq Gold for 9 minutes at 95°C, denaturation for 1 minute at 95°C, annealing for 1 minute at 55°C and extension at 72°C for 1 minute, for a total of 40 cycles, followed by a 5-minute terminal extension step at 72°C. Amplicons were denatured in 0.4 N NaOH. In a reverse-line blot assay, 40 μL of the denatured product were added to 3 mL of hybridization buffer containing the HPV genotypes and 2 concentrations of the β-globin probes, immobilized on nylon strips. Positive hybridization was detected by streptavidin-horseradish peroxidase-mediated colour precipitation on the membrane at the probe line. In specimens that were considered HPV-negative, the 2 β-globin lines (high and low copies) either appeared at levels comparable with those of positive controls or were re-amplified. Only cases that had satisfactory β-globin levels were included in the study. A HPV infection was classified as single and multiple infections. Multiple infections include the association with hr and/or lr-HPV types.

### Cytology

All women had a cervical smear and the results of cervical smears were classified in accordance with the 2001 Bethesda System [14]. Squamous cell abnormalities less severe than invasive carcinoma were classified as atypical squamous cells of undetermined significance (ASC-US) and atypical squamous cells, cannot exclude high-grade intraepithelial lesion (ASC-H), low-grade squamous intraepithelial lesion (LSIL), and high-grade squamous intraepithelial lesion (HSIL). For statistical purposes, ASC-US was grouped with LSIL and ASC-H with HSIL. Endocervical glandular cell abnormalities less severe than invasive adenocarcinoma are classified into atypical glandular cells (AGC), AGC associated with high-grade squamous intraepithelial lesion (AGC-HSIL) and adenocarcinoma *in situ* (AIS).

### Histopathology

Cervical conization specimens, obtained with either Large Loop Excision of the Transformation Zone (LLETZ) or Loop Electrosurgical Excision Procedure (LEEP) were obtained from all patients. The specimens were reviewed according to the World Health Organization (WHO) criteria [15] and were classified as: CIN1, CIN2, CIN3, invasive squamous CC or *in situ* (AIS) and invasive adenocarcinoma. Three-hundred and five (93%) women had squamous and 23 (7%) had glandular lesions. In women with squamous lesions, 82 (25%) had CIN1, 61 (19%) CIN2, 159 (48%) CIN3, and 3 (<1%) had CC. In those with glandular lesions, 13 (4%) had AIS and 10 (3%) had CC. For statistical purposes, CIN 2, CIN3 and CC were grouped as CIN2 or worse (223 cases (68%).

### Statistical analysis

**Figure 1:**
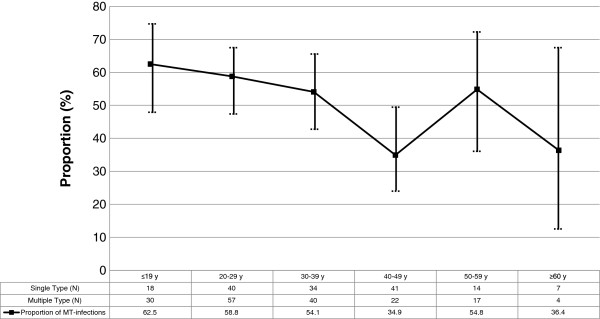
Age stratified proportion of multiple-type HPV infections (95% confidence intervals displayed in vertical bars).

Data were stored in electronic sheets and analysed with the R Environment for Statistical Computing. P < 0.05 was considered significant. Fisher’s Exact tests were used to compare the prevalence of single and multiple HPV infection across histological (CIN1 vs. CIN2 or worse; CIN2 or worse vs. glandular) strata. Chi-squares (and Fisher’s Exact test where appropriate) were used to compare the prevalence of single and multiple HPV infections in different age groups. Two-sided 95% confidence intervals were calculated for the proportion of women with MT infections displayed in Figure 1.

## Results

Table 1 shows cytology results and age strata of the women according to the final pathological diagnoses (histology). For the majority of cases, the glandular and squamous nature of the cytology and final pathological diagnoses converged, as did the severity of the lesion. Thirty-six per cent of the women were 29 years old or younger, 49% were between 30 and 49 years old and 15% were older than 50 years.

**Table 1:** Women distribution according to cytology and age and histology results

		**Squamous lesions**				**Glandular lesions**	
		**(Histology)**				**(Histology)**	
	**TOTAL**	**CIN1**	**CIN2**	**CIN3**	**CC**	**AIS**	**CC**
	**n (%)**	**n (%)**	**n (%)**	**n (%)**	**n (%)**	**n (%)**	**n (%)**
**Cytology**							
Normal	37 (11)	19 (23)	6 (10)	12 (7)	0	0	0
**Squamous lesions**							
ASC-US/LSIL	46 (14)	26 (32)	8 (13)	12 (8)	0	0	0
ASC-H/HSIL	169 (52)	22 (27)	37 (61)	103 (65)	3 (100)	3 (24)	1 (10)
**Glandular lesions**							
AGC	65 (20)	15 (18)	9 (15)	27 (17)	0	8 (60)	6 (60)
AGC-HSIL	6 (2)	0	1 (1)	4 (2)	0	1 (8)	0
AIS	5 (1)	0	0	1 (1)	0	1 (8)	3 (30)
**AGE (years)**							
≤ 19	12 (4)	5 (6)	3 (5)	3 (2)	1 (33)	0	0
20-29	107 (32)	35 (43)	22 (36)	48 (31)	0	1 (8)	1 (10)
30-39	87 (27)	22 (26)	16 (27)	42 (26)	1 (33)	5 (38)	1 (10)
40-49	74 (22)	13 (16)	15 (24)	40 (25)	0	4 (30)	2 (20)
50-59	36 (11)	7 (9)	3 (5)	19 (12)	0	2 (16)	5 (50)
≥ 60	12 (4)	0	2 (3)	7 (4)	1 (33)	1 (8)	1 (10)
**TOTAL**	328 (100)	82 (100)	61 (100)	159 (100)	3 (100)	13 (100)	10 (100)

CIN: cervical intraepithelial neoplasia, CC invasive cervical cancer, AIS: in situ adenocarcinoma, ASC-US/LSIL: atypical squamous cells of undetermined significance/ low-grade squamous intraepithelial lesion, ASC-H/HSIL: atypical squamous cells, cannot exclude high-grade intraepithelial lesion/high-grade squamous intraepithelial lesion, AGC = atypical glandular cells, AGC-HSIL = atypical glandular cells associated with high-grade squamous intraepithelial lesion, AIS = *in situ* adenocarcinoma.

In Table 2, multiplicity of HPV infection is compared as related to the final histology. Two-hundred eighty seven (87%) women had at least one HPV detected and 149 (52%) had MT infections. HPV was not detectable in 26% of CIN1, 10% of CIN2, 5% of CIN3 or cervical cancer and 22% of the glandular lesions. Fifty seven per cent of the women with CIN1 and 53% of those with CIN2 had MT infections; those CIN3 or cervical cancer had the same number of single and multiple infection, when HPV was detectable (p = 0.66). For women with glandular lesions, 13 (72%) had ST and only 5 (28%) had MT infections. Women with squamous lesions had a significantly higher prevalence of MT infections compared to women with glandular lesions (p = 0.04). The most frequent HPV associations were HPV 16/58 (19 cases), 16/52 (12 cases) and 16/18 (10 cases) (data not shown).

**Table 2:** Multiplicity of HPV infection according to histopathology

**HPV infection**	**Squamous lesions**			**Glandular lesions (AIS and CC)**
	**CIN1**	**CIN2**	**CIN3 and CC**	
	**n (%)**	**n (%)**	**n (%)**	**n (%)**
HPV detectable	60 (74)	55 (90)	154 (95)	18 (78)
*Single type (ST)*	26 (43)	22 (40)	77 (50)	13 (72)
*Multiple type (MT)*	34 (57)	33 (60)	77 (50)	5 (28)
HPV not detectable	22 (26)	6 (10)	8 (5)	5 (22)
TOTAL	82 (100)	61 (100)	162 (100)	23 (100)

CIN: cervical intraepithelial neoplasia, CC: invasive cervical câncer, AIS: in situ adenocarcinoma. HPV infections, whether single type or multiple type, did not differ in women with CIN1 or CIN2 or worse (p = 0.66). The prevalence of MT infection was significantly higher in women with CIN2 or worse compared to those with glandular lesions (p = 0.04).

Table 3 shows the HPV types in ST and MT infections. HPV16 was detected in 142 cases (49% of all HPV positive cases) and topped the prevalence rank in both ST and MT groups. Infection by HPV58, 52, 31, 35 and 33 (all of them from alpha-9 species) followed. HPV18 was detected in only 23 cases (8% of all hr-HPV positive cases). ST HPV infections were less prevalent than their MT counterparts containing the same HPV type.

**Table 3:** HPV types in single and multiple infections

**HPV type**	**ST infection**	**MT infections**	**TOTAL**
	**n (%)**	**n (%)**	**n**
hr-HPV			
16	61 (43)	81 (57)	142
58	12 (27)	33 (73)	45
52	9 (26)	25 (74)	34
31	11 (34)	21 (66)	32
35	6 (22)	21 (78)	27
33	7 (26)	20 (74)	27
18	4 (17)	19 (83)	23
51	3 (14)	18 (86)	21
45	3 (21)	11 (79)	14
56	2 (20)	8 (80)	10
39	2 (20)	8 (80)	10
Other hr-HPV	10 (17)	49 (83)	59
Any hr-HPV	130 (47)	148 (53)	278
lr-HPV			
6	3 (27)	5 (63)	8
11	1 (100)	0	1
Other lr-HPV	4 (8)	48 (92)	52
Any lr-HPV	8 (13)	53 (87)	61
Any HPV	138 (48)	149 (52)	287

All HPV types from single to multiple infections were computed individually, for% calculation the denominator is represented by the total number of infections (single or multiple) by type. From alfa-9 HPV group: 16, 31, 33, 35, 52 and 58. From alfa-7 HPV group: 18, 39 and 45. ST: single type, MT: multiple type, hr-HPV: high risk HPV, lr-HPV: low-risk HPV.

Table 4 compares ST and MT statuses of the most prevalent HPV types according to histology. MT infections with either or both HPV16 and 18 were significantly more prevalent in women with squamous than in those with glandular lesions (p = 0.04 and 0.03 respectively). Although the majority of CIN2 or worse were associated with hr-HPV, six cases were associated with lr-HPV types only. All glandular lesions were associated with HPV16 and/or HPV18 (p = 0.04 and p = 0.03, respectively), 13 of these in ST configuration. Only five glandular lesions had MT HPV infection associated with them.

**Table 4:** Single and multiple type HPV infection status according to histopathological diagnoses

**HPV type**	**Type of infection**	**CIN1**	**CIN2 or worse**	**Glandular (AIS and CC)**	**P1**	**P2**
		**(n = 60)**	**(n = 209)**	**(n = 18)**	**CIN 1 vs CIN 2 or worse**	**CIN 2 or worse vs glandular**
		**N(%)**	**N(%)**	**N(%)**		
hr-HPV						
16	Single	6 (10)	45 (22)	10 (55)		
	Multiple	13 (22)	64 (31)	4 (22)	0.46	0.04
58	Single	0	12 (6)	0		
	Multiple	8 (13)	24 (11)	1 (5)	0.08	1
52	Single	0	9 (4)	0		
	Multiple	5 (8)	19 (9)	1(5)	0.29	1
31	Single	4 (7)	7 (3)	0		
	Multiple	5 (8)	16 (8)	0	0.68	NC
35	Single	2 (3)	4 (2)	0		
	Multiple	7 (12)	13 (6)	1 (5)	1	0.98
33	Single	1 (<2)	6 (3)	0		
	Multiple	2 (3)	17 (8)	0	1	NC
18	Single	1 (<2)	0	3 (17)		
	Multiple	2 (3)	3 (<2)	4 (22)	NC	0.03
51	Single	1	2 (1)	0		
	Multiple	7 (12)	10 (5)	1 (5)	1	1
45	Single	0	3 (<2)	0		
	Multiple	2 (3)	8 (4)	1 (5)	1	NC
56	Single	0	2 (1)	0		
	Multiple	3 (5)	6 (3)	0	1	NC
39	Single	1 (<2)	1 (<1)	0		
	Multiple	3 (5)	5 (2)	0	1	NC
Other	Single	6 (10)	4 (2)	0		
hr-HPV	Multiple	13 (22)	34 (16)	1 (5)	0.07	1
Any hr-	Single	22 (37)	95 (45)	13 (72)		
HPV	Multiple	34 (57)	110 (53)	5 (28)	0.36	<0.05
lr-HPV						
6	Single	2 (3)	1 (<1)	0		
	Multiple	2 (3)	3 (<2)	0	1	NC
11	Single	0	1 (<1)	0		
	Multiple	0	0	0	NC	NC
Others	Single	6 (10)	4 (2)	0		
lr-HPV	Multiple	15 (25)	34 (16)	1 (5)	0.14	NC
Any HPV	Single	26 (43)	99 (47)	13 (72)		
	Multiple	34 (57)	110 (53)	5 (28)	0.66	0.04

Prevalence was calculated by dividing the number of infected cases per HPV type by the number of women with the histopathological diagnosis. Totals surpass 100% because women with MT infections are counted at least twice. hr-HPV: high-risk HPV, lr-HPV: low-risk HPV, CIN: cervical intraepithelial neoplasia, AIS: in situ adenocarcinoma, CC: invasive cervical cancer.

Figure 1 shows the proportion of MT HPV infections as related to patient age. MT infections are the most prevalent in women <29 years of age. At the strata age of 40–49 years threshold, the prevalence of ST and MT swaps in favour of ST infections. Another shift occurs at 50–59 years threshold, but the prevalence of MT infections is reduced again in older (≥60 years) women.

## Discussion

In this study on 328 Brazilian women with pre-invasive and invasive squamous and glandular lesions, 43% of the women were detected with HPV16, but only 18.5% of the women had HPV16 as ST infection. HPV16 was followed in prevalence by other alpha-9 HPV types, and HPV18 appeared only in the seventh position. Women with squamous lesions had a higher proportion of MT infections (most frequent associations were HPV 16/52, 16/58 and 16/18) compared to their counterparts with glandular lesions, despite the small number of glandular samples. Women with glandular lesions, by their turn, most likely had ST HPV16 or 18. A bimodal prevalence curve for MT infections shows prevalence peaks at a very young (<29 years) age and in women 50–59 years of age.

In this mostly urban study, HPV infection with HPV16, followed by HPV58, 52, 31, 35 and 33, were typical. The most common HPV types are essentially from the alpha-species, which are the most frequently detected types in women with squamous lesions. Simonella *et al.*[16] found similar trends in New Zealander women with CIN2 or worse aged 20–69 years, for whom HPV16 was the most prevalent HPV type detected (found in 44.1% of the women) followed by HPV52 (16.8%), HPV31 (15.2%), HPV33 (11.8%), HPV18 (11.3%) and HPV58 (10.1%). Quint *et al.*[17] found that the most common HPV type in women with CIN3 was HPV16, accounting for 56.9% of ST and MT infections. This type followed by HPV31 (10.0%), 52 (8.4%) and 18 (4.6%). In their study, only two CIN3 samples were positive for any single lr-HPV: HPV6 was detected in both cases as a ST infection. Kirschner *et al.*[18] studied 266 Danish women with CIN2 and/or CIN3 and seven with AIS. In that sample, 77.9% of the women were diagnosed with a ST infection. The most prevalent HPV types were: HPV16, detected >50% of the sample, followed by HPV33, 31, 18 and 52. Only 21.4% were diagnosed with MT infections. We could identify that the prevalence of HPV types in women with squamous cervical lesions differs from that in women with normal cervix. In a previous study with 1,509 Brazilian healthy young women without cervical lesions of five different centres, aged 15 to 25 years, we found an overall prevalence of HPV of 29.7%. The most prevalent HPV type was HPV16, followed by HPV51, 52, 31 and 68 [19]. Among 8,656 healthy Danish women aged 20 to 29 years, the prevalence of HPV types in decreasing rank was HPV16 followed by HPV31, 52, 51, 33, 39, 45 and 56 [10]. In their study, women infected by alpha-9 HPV species were at a higher risk of developing CIN2 or worse lesion during a 10-year follow-up period [10].

In our study, squamous lesions were associated with a higher prevalence of MT infections compared to women with glandular lesions. It remains largely unknown whether MT infections are associated with a higher risk of developing squamous cervical lesions. In our study, in spite of the limited sample size, we found no difference in ST and MT prevalence comparing women with CIN1 with those with CIN2 or worse. By contrast, Pista *et al.*[12], studying Portuguese women, found a significant association between MT infections and disease severity. Argyri *et al.*[11] also found that MT infections were significantly more common in women with squamous intraepithelial lesions compared with healthy women. In another study with women undergoing colposcopy for ASCUS/LSIL, Balbi *et al*. (2012) [20] concluded that the infection with multiple HPV types is a significant risk factor for high-grade CIN.

Simultaneous presence of multiple HPV genotypes may be associated with a increased risk of high-grade squamous lesions or invasive cancer than the presence of single-type HPV infection. The classic study from Trottier *et al*. (2006) [21] showed that infections with multiple HPV may synergistically boost carcinogenesis. In a recently published Brazilian study co-infecting HPV genotypes occurred in a high proportion of sexually active adolescents. Socio-demographic or sexual behaviour factors associated with single HPV infection were similar to those associated with multiple HPV types. There was a higher risk of cytological abnormalities in women infected with multiple HPV types suggesting a potential role of co-infection in the natural history of HPV infection [22]. However, in another relatively recent study [23], the difference between the duration of single and that of concurrent multiple type-specific prevalent HPV infections was not significant. Concurrent, prevalent detection of additional HPV types did not change the likelihood of viral persistence.

In our sample, glandular lesions were associated essentially with HPV16 or 18 ST infections. Quint *et al.*[17] also detected ST HPV16 or HPV18 infections in excess of 70% of women with AIS or glandular-type CC. In the series, HPV35 and 45 accounted for one case of AIS (3.0%) and 10.9% of CC. A meta-analysis from 2007 showed that HPV16 followed by 18 were the most common types in all continents. However, in Africa, Asia and South/Central America, this preponderance is not as marked as in Europe, North America and Australia. The next most common HPV types were the same in each continent, namely HPV31, 33, 35, 45, 52 and 58, although their relative importance differed somewhat by region [24]. In our study, HPV 58 and 52 were more frequently detected than the other types, including HPV 18 which ranked only 7^th^. In the metanalysis [24], HPV18 was significantly more prevalent in adeno/adenosquamous carcinoma than in squamous cell carcinoma, with the reverse being true for HPV16, 31, 33, 52 and 58. In our sample, stand-alone infection by HPV 16 or 18 were responsible for most of the glandular lesions.

Another recent study examined the prevalence of HPV type distribution in Brazilian women with and without cervical lesions. From a total of 132 women with amplified HPV-DNA the frequency of HPV HR-types was 75%, with top appearing types being HPV16 (28%), followed by HPV18 (14.4%), HPV45 (7.6%), HPV58 (6.8%), HPV66 (6.8%), HPV31 (3.8%) and HPV33 (3.0%). The mean age of the 132 women were 39.5 years, ranging from 25 to 59 years of age. A significantly lower proportion of LSIL and HSIL was found among women infected by HPV16 and/or HPV18 when compared to the ones infected by other HR-types. Their data also showed that infection by alpha-7 and alpha-9 species presented a significant distinct distribution by age at diagnosis respective to women positive for other HPV types. However, separate comparisons among alpha-7 infections, alpha-9 infection, and infections by other HPV types, did not show a significant different distribution by age [25]. Another approach to the question was attempted by Fernandes *et al*. (2010) who examined the HPV prevalence in archival samples obtained from patients with cervical pre-malignant and malignant lesions from Northeast Brazil. In that study, among the HPV-positive samples, 86.7% had a single infection, while 13.3% had a double infection. These findings contrast with ours, since we detected a higher prevalence of MT infections in all lesion groups [26].

Our results may bear significance in the era of prophylactic HPV vaccination. Both bivalent and quadrivalent prophylactic HPV vaccines target hr-HPV16 and 18 and had demonstrated different cross protection against HPV31, 33, 45, 52 and 58, potentially diverting 90% of cervical cancer risk. In addition to a new nonavalent vaccine against HPV16, 18, 31, 33, 45, 52 and 58 - now in phase 3 testing - protection against the most prevalent HPV types is in sight [27].

The main strength of this study is that all women had a complete histological evaluation, giving to our sample a strong reliability related to the gravity of the cervical lesion. We also took care to assess all known HPV types using reverse line blot hybridization assay, which is an accurate and sensitive method. On the other hand, the sample is modest in size, especially women with glandular lesions, which unfortunately is a common situation in most recent papers on the subject.

## Conclusions

Collectively, our data indicate that prevention strategies for pre-invasive and invasive squamous lesions should be focused on HPV16 and a few alpha-9 HPV types. It is clear to us that in young women, prophylaxis must cover a large amalgam of HPV types beyond classic HPV16 and 18. For the prevention of glandular lesions, ST HPV16 or 18 infections should be addressed, with other alpha-9 or alpha-7 HPV types being of marginal importance.

## Competing interests

The authors declare that they have no competing interests.

## Authors’ contributions

LSAR, SHRS and SD designed the study and wrote the manuscript. SD coordinated the study group. LOS analysed the data and wrote the manuscript. RRFA and AAR assisted with the experiments. LOS, LCZ and SD revised the manuscript. All authors read and approved the final version of the manuscript.

## Pre-publication history

The pre-publication history for this paper can be accessed here:

http://www.biomedcentral.com/1471-2334/14/214/prepub

## References

[B1] IARC – International Agency for Research on Cancer. World Health Organization Globocan 2008. Planning and Implementing Cervical Cancer Prevention and Control Programs – A manual for managers. Alliance for Cervical Cancer Prevention 2008 http://screening.iarc.fr/manual/ACCP_screen.pdf

[B2] INCA – Instituto Nacional do Câncer Ministério da Saúde. Coordenação nacional de prevenção e vigilância do câncer. Incidência do câncer no Brasil 2012 http://www.inca.gov.br/estimativa/2014/

[B3] Zur HH **Papillomaviruses and cancer: from basic studies to clinical applicatio**n Nat Rev Cancer 2002 14 5 342 350 10.1038/nrc798 12044010

[B4] Schiffman M Castle PE Human Papillomavirus: epidemiology and public health Arch Pathol Lab Med 2003 14 8 930 934 12873163 10.5858/2003-127-930-HPEAPH

[B5] Chen HC You SL Hsieh CY Schiffman M Lin CY Pan MH Chou YC Liaw KL Hsing AW Chen CJ CBCSP-HPV Study Group Prevalence of genotype-specific human papillomavirus infection and cervical neoplasia in Taiwan: a community-based survey of 10,602 women Int J Cancer 2011 14 5 1192 1203 10.1002/ijc.25685 20853317

[B6] Khan S Jaffer NN Khan MN Rai MA Shafiq M Ali A Pervez S Khan N Aziz A Ali SH Human papillomavirus subtype 16 is common in Pakistani women with cervical carcinoma Int J Infect Dis 2007 14 4 313 317 10.1016/j.ijid.2006.06.007 17291804

[B7] Mirabello L Schiffman M Ghosh A Rodriguez AC Vasiljevic N Wentzensen N Herrero R Hildesheim A Wacholder S Scibior-Bentkowska D Burk RD Lorincz AT Elevated methylation of HPV16 DNA is associated with the development of high grade cervical intraepithelial neoplasia Int J Cancer 2013 14 6 1412 1422 10.1002/ijc.27750 22847263 PMC3493709

[B8] Pantawala IY Bauer HM Miyamoto J Park IU Huchko MJ Smith-McCune KK A systematic review of randomized trials assessing human papillomavirus testing in cervical cancer screening Am J Obstet Gynecol 2013 14 5 343 353 10.1016/j.ajog.2012.11.013 23159693 PMC3686555

[B9] Schiffman M Castle PE Jeronimo J Rodriguez AC Wacholder S Human Papillomavirus and cervical cancer Lancet 2007 14 9590 890 907 10.1016/S0140-6736(07)61416-0 17826171

[B10] Kjær SK Frederiksen K Munk C Iftner T Long-term absolute risk of cervical intraepithelial neoplasia grade 3 or worse following human papillomavirus infection: role of persistence J Natl Cancer Inst 2010 14 19 1478 1488 10.1093/jnci/djq356 20841605 PMC2950170

[B11] Argyri E Papaspyridakos S Tsimplaki E Michala L Myriokefalitaki E Papassideri I Daskalopoulou D Tsiaoussi I Magiakos G Panotopoulou E A cross sectional study of HPV type prevalence according to age and cytology BMC Infect Dis 2013 14 53 10.1186/1471-2334-13-53 23363541 PMC3575232

[B12] Pista A de Oliveira CF Cunha MJ Paixao MT Real O Prevalence of human papillomavirus infection in women in Portugal: the CLEOPATRE Portugal Study Int J Gynecol Cancer 2011 14 6 1150 1158 10.1097/IGC.0b013e31821dd3b2 21792018

[B13] Wentzensen N Sun C Ghosh A Kinney W Mirabello L Wacholder S Shaber R LaMere B Clarke M Lorincz AT Castle PE Schiffman M Burk RD Methylation of HPV18, HPV31, and HPV45 genomes and cervical intraepithelial neoplasia grade 3 J Natl Cancer Inst 2012 14 22 1738 1749 10.1093/jnci/djs425 23093560 PMC3571257

[B14] Nayar R Solomon D Second edition of The Bethesda System for reporting cervical cytology – atlas, website, and Bethesda interobserver reproducibility project CytoJournal 2004 14 1 4 10.1186/1742-6413-1-4 15504231 PMC526759

[B15] Scully RE Bonfiglio TA Kurman RJ Silverberg SG Wilkins EJ Histological Typing of Female Genital Tract Tumors World Health Organization. International Histological Classification of Tumors 1994 2 Berlin: Springer-Verlag 36 49

[B16] Simonella LM Lewis H Smith M Neal H Bromhead C Canfell K Type-specific oncogenic human papillomavirus infection in high grade cervical disease in New Zealand BMC Infect Dis 2013 14 114 10.1186/1471-2334-13-114 23452957 PMC3607885

[B17] Quint KD de Koning MN van Doorn LJ Quint WG Pirog EC HPV genotyping and HPV16 variant analysis in glandular and squamous neoplastic lesions of the uterine cervix Gynecol Oncol 2010 14 2 297 301 10.1016/j.ygyno.2010.02.003 20207397

[B18] Kirschner B Schledermann D Holl K Rosenlund M Raillard A Quint W Molijn A Jenkins D Junge J HPV-genotypes in high-grade intraepithelial cervical lesions in Danish women Acta Obstet Gynecol Scand 2013 14 9 1032 1040 10.1111/aogs.12162 23647074

[B19] Roteli-Martins CM de Carvalho NS Naud P Teixeira J Borba P Derchain S Tyring S Gall S Diaz A Blatter M Shier RM Romanowski B Quint WG Issam J Galindo C Schuind A Dubin G Prevalence of human papillomavirus infection and associated risk factors in young women in Brazil, Canada, and the United States: a multicenter cross-sectional study Int J Gynecol Pathol 2011 14 2 173 184 21293281 10.1097/PGP.0b013e3181f38dfe

[B20] Balbi G Napolitano A Giordano F Capuano S Manganaro MA Di Martino L Fusco D Grauso F Seguino E Role of the association of high-risk HPV identified by real-time PCR in cervical preneoplastic lesions Eur J Gynaecol Oncol 2012 14 5 467 471 23185789

[B21] Trottier H Mahmud S Costa MC Sobrinho JP Duarte-Franco E Rohan TE Ferenczy A Villa LL Franco EL Human papillomavirus infections with multiple types and risk of cervical neoplasia Cancer Epidemiol Biomarkers Prev 2006 14 7 1274 1280 10.1158/1055-9965.EPI-06-0129 16835323

[B22] Figueiredo Alves RR Turchi MD Santos LE Guimarães EM Garcia MM Seixas MS Villa LL Costa MC Moreira MA Alves MD Prevalence, genotype profile and risk factors for multiple human papillomavirus cervical infection in unimmunized female adolescents in Goiania, Brazil: a community-based study BMC Public Health 2013 14 1 1041 10.1186/1471-2458-13-1041 24188572 PMC3819257

[B23] Campos NG Rodriguez AC Castle PE Herrero R Hildesheim A Katki H Kim JJ Wacholder S Morales J Burk RD Schiffman M Persistence of concurrent infections with multiple human papillomavirus types: a population-based cohort study J Infect Dis 2011 14 823 827 10.1093/infdis/jiq131 21257737 PMC3071138

[B24] Smith JS Lindsay L Hoots B Keys J Franceschi S Winer R Clifford GM Human papillomavirus type distribution in invasive cervical cancer and high-grade cervical lesions: a meta-analysis update Int J Cancer 2007 14 3 621 632 10.1002/ijc.22527 17405118

[B25] Oliveira-Silva M Lordello CX Zardo LM Bonvicino CR Moreira MA Human Papillomavirus in Brazilian women with and without cervical lesions Virol J 2011 14 4 10.1186/1743-422X-8-4 21208414 PMC3024957

[B26] Fernandes JV Meissner RV Carvalho MG Fernandes TA Azevedo PR Sobrinho JS Prado JC Villa LL Prevalence of human papillomavirus in archival samples obtained from patients with cervical pre-malignant and malignant lesions from Northeast Brazil BMC Res Notes 2010 14 1 96 10.1186/1756-0500-3-96 20377903 PMC2857859

[B27] Van de Velde N Boily MC Drolet M Franco EL Mayrand MH Kliewer EV Coutlée F Laprise JF Malagón T Brisson M Population-level impact of the bivalent, quadrivalent, and nonavalent human Papillomavirus Vaccines: a model-based analysis J Natl Cancer Inst 2012 14 1712 1723 10.1093/jnci/djs395 23104323

